# Determination of Stable Reference Genes for Gene Expression Analysis in Black Rockfish (*Sebastes schlegeli*) Under Hypoxia Stress

**DOI:** 10.3390/genes16010009

**Published:** 2024-12-25

**Authors:** Xiatian Chen, Yujie Yu, Tao Gao, Zhifei Liu, Shuaiyu Chen, Yudong Jia

**Affiliations:** Yellow Sea Fisheries Research Institute, Chinese Academy of Fishery Sciences, Qingdao 266071, China; chenxiatian1216@163.com (X.C.); yyjie0929@163.com (Y.Y.); gaotao0098@hotmail.com (T.G.); lzf559900@163.com (Z.L.); shoushuaiyuchen@163.com (S.C.)

**Keywords:** dissolved oxygen, fish, reference gene, RT-qPCR, stability

## Abstract

Background: Hypoxia triggers stress, leading to significant alterations in gene expression patterns, which in turn affect fish’s growth and development. Real-time quantitative PCR (RT-qPCR) is a pivotal technique for assessing changes in gene expression. However, its accuracy is highly contingent upon the stable expression of reference genes. *Ribosomal RNA* (*18s*), *β-actin* (*actb*), *elongation factor 1-α* (*ef1a*), *α tubulin* (*tuba*), and *ribosomal protein L17* (*rpl17*) are the widely used reference genes, but their expression stability in the tissues of black rockfish under hypoxic conditions remains unclear. Methods: The expression of genes was detected by RT-qPCR and the stability was assessed by Delta Ct, geNorm, NormFinder, and BestKeeper algorithms. Results: Results showed that *tuba* exhibited stable expression in liver, heart, gill tissues under normoxic conditions, and in the liver and head kidney under hypoxic conditions. *Ef1a* was identified as the most stably expressed gene in gill tissue under hypoxia. For hypoxic heart studies, *rpl17* and *tuba* were recommended as reference genes. *18s* showed high stability in spleen tissue under hypoxic conditions. *Actb* was the most stably expressed gene in spleen and head kidney tissues under normoxic conditions. Conclusions: The identified reference genes exhibited tissue-specific stability, and it was necessary to select appropriate reference genes based on the specific tissue type for gene expression studies under hypoxic conditions. These findings help in enhancing the accuracy of gene expression analysis in the mechanism of hypoxia for black rockfish.

## 1. Introduction

On account of the rise in global temperatures due to human activities, coupled with the prevalence of high cultivation density in aquaculture, the content of dissolved oxygen (DO) is lower than the normal level [1,2]. DO is a critical and potentially problematic abiotic factor in aquaculture [3,4]. Insufficient dissolved oxygen levels in water initially exert direct adverse effects on aquatic animals. Subsequently, it indirectly impacts the aquatic environment through other organisms, inflicting varying degrees of harm on their growth, reproduction, and even survival. Hypoxia disrupts numerous intracellular processes, leading to alterations in tissue morphology and function. However, our understanding of this phenomenon remains incomplete [5,6,7]. Gene expression studies offer a valuable approach to gaining a deeper insight into the complex regulatory networks that govern the hypoxic response. Constructed in 1996, RT-qPCR introduced fluorescent labels to the PCR reaction system, utilizing the accumulation of fluorescence signals to monitor the entire PCR process, rendering each cycle “visible” [8,9]. Up to now, RT-qPCR remains the most sensitive and precise method for determining DNA copy numbers in samples and is an indispensable tool in molecular biology research [10].

In 2009, Bustin et al. formulated guidelines for RT-qPCR experimental protocols, providing researchers with critical information to ensure the feasibility, precision, accurate interpretation, and reproducibility of their experiments [11]. The selection of a useful reference gene is paramount in RT-qPCR experiments, as it must exhibit consistent expression across the experimental system. However, given the intricacies of biological systems, no single reference gene is universally applicable. Consequently, for an accurate assessment of gene expression changes, proper normalization is indispensable. The most commonly utilized reference genes are those that exhibit stable expression, such as housekeeping genes. These genes are persistently active across all cells and tissues, playing an essential role in sustaining the fundamental life processes of cells, with their expression typically remaining unaffected by environmental factors [12,13]. *Actb*, *ef1a*, *tuba*, *rpl17*, and *18s* were often employed as reference genes, and in previous studies, we briefly compared the stability of these genes in the liver of black rockfish [6,14].

Black rockfish is the main marine fish species widely cultured in offshore sea cages in northern China, Korea, and Japan due to its tender meat and economic values [15,16,17]. At present, there are many studies on the black rockfish, mainly focusing on environmental stress and disease resistance [18,19,20,21]. Ma et al. have identified the *ef1a*, *rpl17*, and *actb* were the appropriate reference genes in larvae development stages of black rockfish [22]. In addition, Jin et al. reported that *rab10*, *elf5a1*, *pairb*, and *btf3l4* were more stable genes in black rockfish which were reared in normal conditions [23]. However, in terms of research, the gene expression patterns of the heart indicates its functional state and its reactions to diseases or shifts in the environment [24]. The liver, a key player in energy metabolism, substance synthesis, and immune responses, is crucial for understanding metabolic and detoxification processes [25]. The spleen and kidneys, being pivotal immune organs, are instrumental in gauging the body’s defense mechanisms [26]. In the case of aquatic organisms, the gills, which are essential for respiration, offer insights into their adaptability to aquatic environmental changes and the intricate mechanisms of respiration and osmotic pressure regulation through their gene expression profiles [27]. Currently, there is lack of analysis identifying reference genes suitable for hypoxia conditions in various tissues of black rockfish.

In this study, *18s*, *actb*, *ef1a*, *tuba*, and *rpl17* were selected, and their expression in the heart, liver, gill, spleen, and head kidney of black rockfish under the hypoxia conditions were detected. Furthermore, we analyzed the stability in these tissues using the Delta Ct, geNorm, NormFinder, and BestKeeper methods. These results could provide reliable methods for RT-qPCR detection and improve the accuracy of target gene expression in hypoxia research of black rockfish.

## 2. Materials and Methods

### 2.1. Experimental Approach and Sample Collecting

A total of thirty-six juvenile black rockfish with an average body length of 21.00 ± 6.00 cm and a body weight of 88.01 ± 5.34 g were obtained from Tianyuan Aquaculture Co., Ltd., Yantai, Shandong, China. The fish were reared in experimental tanks, each with a capacity of 90 L, for 2 weeks each. During the experimental period, the fish were fed twice daily at 9:00 A.M. and 4:00 P.M. The aquaculture water conditions were maintained at a temperature of 18 °C, salinity of 30, pH value of 7.85, DO content of 8.73 ± 0.36 mg/L, and ammonia nitrogen concentration of less than 0.1 mg/L. The critical oxygen partial pressure (pcrit) is defined as the threshold below which an animal’s standard metabolic rate can no longer be sustained [28]. In a previous study, we determined experimentally the pcrit and loss of equilibrium (loe) for black rockfish using a dissolved oxygen meter (Cat#: AZ86031, Xin Heng, Shanghai, China) to automatically monitor and record changes in dissolved oxygen levels in the water [6].

Nine fish were randomly selected and placed into culture buckets, with each group consisting of three buckets. We established control, pcrit, loe, and recovery groups, with durations of 24 h. A closed static water method was used to create a hypoxic environment, where the culture buckets for the treatment groups were sealed to prevent water exchange with the air. When all fish were unable to maintain balance, the hypoxia treatment was terminated, and air and water exchange was immediately initiated. Within 20 min, the dissolved oxygen in the water returned to normal levels and was maintained for 24 h. After the experiment, fish were anesthetized with an overdose of tricaine methane sulfonate (200 mg/L) (Cat#: A5040, Sigma, St Louis, MO, USA), and the heart, liver, gills, spleen, and kidney tissues were collected using scissors and forceps, then stored in liquid nitrogen.

### 2.2. RNA Isolation and Reverse Transcription

RNA was isolated using Trizol reagent (Cat#: R401, Vazyme, Nanjing, China) according to the instructions. In brief, an appropriate volume of Trizol was added to the crushed tissue block, thoroughly mixed, and then chloroform was added at a ratio of one-fifth that of the Trizol reagent. The mixture was vigorously shaken up and down for 5 s and incubated on ice for 10 min. Subsequently, the sample was spun at 12,000 rpm for 10 min at 4 °C. The supernatant was carefully extracted, and an equal volume of isopropanol was added, mixed well, and incubated on ice for an additional 10 min. Afterward, the mixture was centrifuged at 12,000 rpm for 10 min at 4 °C. The supernatant was removed, and the RNA pellet was washed twice with 75% ethanol, followed by centrifugation at 10,000 rpm for 5 min at 4 °C. Once the pellet was dried, it was suspended in appropriate volume of RNase-free H_2_O. The concentration and quality of RNA were determined through Nanodrop (Thermo Fisher Scientific, Waltham, MA, USA). RNA (OD260/280 = 1.8–2.0, OD260/230 ≥ 2.0, RIN ≥ 8, and total RNA > 10 μg) were used for subsequent experiments. For cDNA synthesis, 1 µg of RNA was reverse-transcribed following the manufacturer’s instructions for the RNA Reverse Transcription Kit (Cat#: R323, Vazyme, Nanjing, China). The resulting cDNA was used for downstream applications.

### 2.3. RT-qPCR

The RT-qPCR experiment was performed using a SYBR Green mix kit (Cat#: Q711, Vazyme, Nanjing, China) according to the instructions. The 20 µL reaction system comprised 1X ChamQ Universal SYBR qPCR mix reagent, 200 nM of each upstream and downstream primer, and 10 ng of cDNA, and H_2_O. After preparing the reaction mixture, the RT-qPCR was conducted on a Quantstudio 5 system (Thermo Fisher Scientific, Waltham, MA, USA). The thermal cycling conditions were as follows: initial denaturation at 95 °C for 30 s, followed by 40 cycles of 95 °C for 10 s, 60 °C for 30 s, and a melting curve analysis consisting of 95 °C for 15 s, 60 °C for 60 s, and 95 °C for 15 s. Each experiment was conducted in triplicate and gene expression levels were determined using the 2^−ΔΔCt^ method. The gene sequences were searched and downloaded from the NCBI database (https://www.ncbi.nlm.nih.gov/, 16 August 2024), and primers were designed using Primer 5 software. The efficiency of primer pairs all exceeded 95%. Primer synthesis was carried out by Beijing Qingke Biotechnology Co., Ltd., Beijing, China. The information on primers is listed in Table 1.

### 2.4. Statistical Analysis

All the experiments were conducted three times, and all results are presented as mean ± standard deviation (SD). The stability of gene expression was assessed by Delta Ct method [29], geNorm (Version 3.4), NormFinder (Version 0.93), and BestKeeper (Version 1) software. The geNorm software assessed the stability of gene by calculating the M value of each internal reference gene [30]. The NormFinder method calculates the suitability of reference genes based on stability values, with lower values indicating more stable expression [31]. BestKeeper is a software program embedded in an EXCEL spreadsheet that evaluates gene stability by calculating and analyzing the correlation coefficient (r) and SD for each gene [32]. The comprehensive assessment on the stability of candidate was performed on the RefFinder website [33].

## 3. Results

### 3.1. Analysis of Primer Amplification

**Figure 1:**
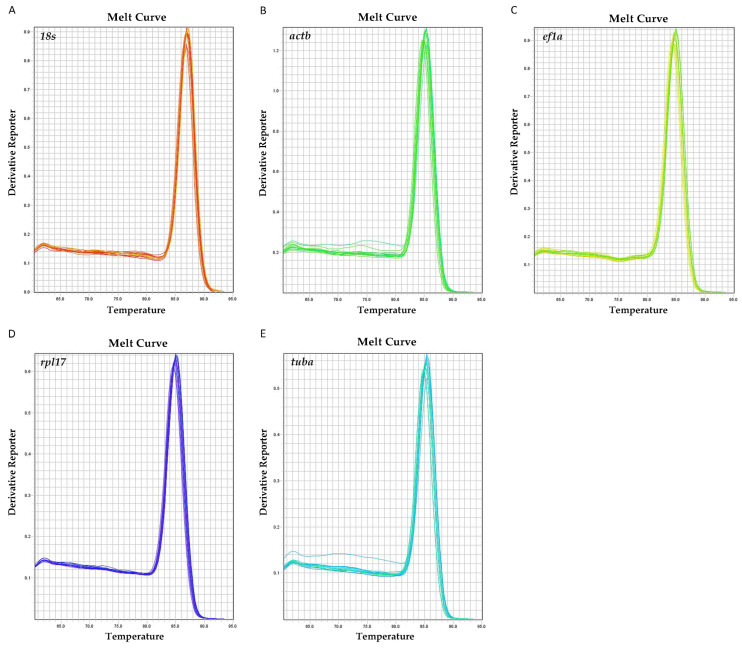
Melt curve analysis of primers. (**A**) Melt curve of *18s* in the sample. (**B**) Melt curve of *actb* in the sample. (**C**) Melt curve of *ef1a* in the sample. (**D**) Melt curve of *rpl17* in the sample. (**E**) Melt curve of *tuba* in the sample. X axis shows the dissolution temperature and the Y axis indicates the derivative report.

The information on primers is presented in Table 1. Melting curve analysis showed that all primers had a single peak after 40 cyclic amplifications, those results showed the high specificity of the primers and validated the suitability for use in RT-qPCR (Figure 1).

### 3.2. Reference Gene Expression Analysis

**Figure 2:**
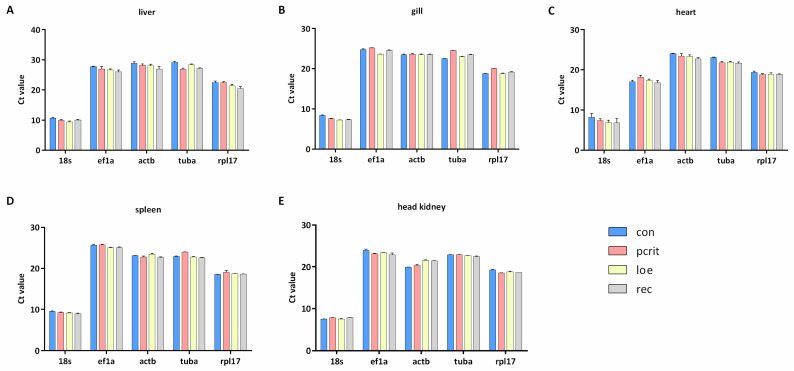
Mean Ct values of candidate reference genes across the experimental groups. (**A**) Ct values of five genes in the liver. (**B**) Ct values of five genes in the gill. (**C**) Ct values of five genes in the heart. (**D**) Ct values of five genes in the spleen. (**E**) Ct values of five genes in the head kidney. Data were expressed as the mean ± SD. con, control; pcrit, critical oxygen level; loe, loss of equilibrium; rec, recovery group.

Ct values are the indicative factors of gene expression in the samples. The distributions of Ct values for each gene in all tissues significantly varied (Figure 2). The value of *18s* was lower in all tissues than other genes. *Tuba*, *actb*, and *ef1a* showed higher expression levels with an average Ct value > 25 in the liver tissue. In the gill tissue, *ef1a* has the highest expression level, followed by *actb*, *tuba*, and *rpl17*. The expression of *actb* was highest in the heart, and the Ct value of *tuba* and *rpl17* all exceeded 20. In the spleen, the reference genes in descending order of Ct value were *ef1a*, *actb*, *tuba*, *rpl17*, and *18s*. *Ef1a* and *tuba* showed higher Ct values (average Ct > 20) in the head kidney, *actb* has a mean Ct value of 19.88, and *rpl17* has a mean Ct value of 19.24.

### 3.3. Expression Stability Analysis by Delta Ct

**Figure 3:**
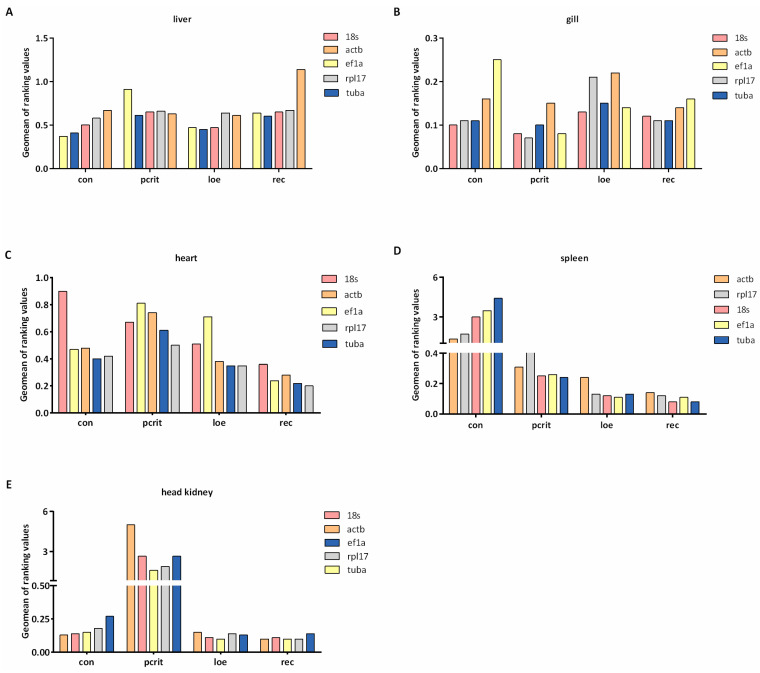
Delta Ct analysis of candidate reference genes’ expression stability. (**A**) The ranking values of five candidate reference genes in the liver. (**B**) The ranking values of five candidate reference genes in the gill. (**C**) The ranking values of five candidate reference genes in the heart. (**D**) The ranking values of five candidate reference genes in the spleen. (**E**) The ranking values of five candidate reference genes in the head kidney. The lower ranking values indicate more stable expression. con, control; pcrit, critical oxygen level; loe, loss of equilibrium; rec, recovery group.

Delta Ct method assesses gene stability by calculating the SD of the differences in Ct values. As shown in Figure 3, we found the most consistent gene under the hypoxia condition was *tuba* in the liver. In the gill, the stable gene was *18s* in the con and loe groups, and the most stable genes were *rpl17* and *18s* in the pcrit group. In heart tissue, *rpl17* was the common stable gene under the hypoxia and control condition. *18s*, *ef1a*, and *tuba* were the most consistent genes in the spleen under the hypoxia, but *actb* was the most stable in con group. For the result in the liver, the most stable gene was *tuba* in the head kidney, followed by *18s* and *rpl17*.

### 3.4. Expression Stability Analysis by geNorm

**Figure 4:**
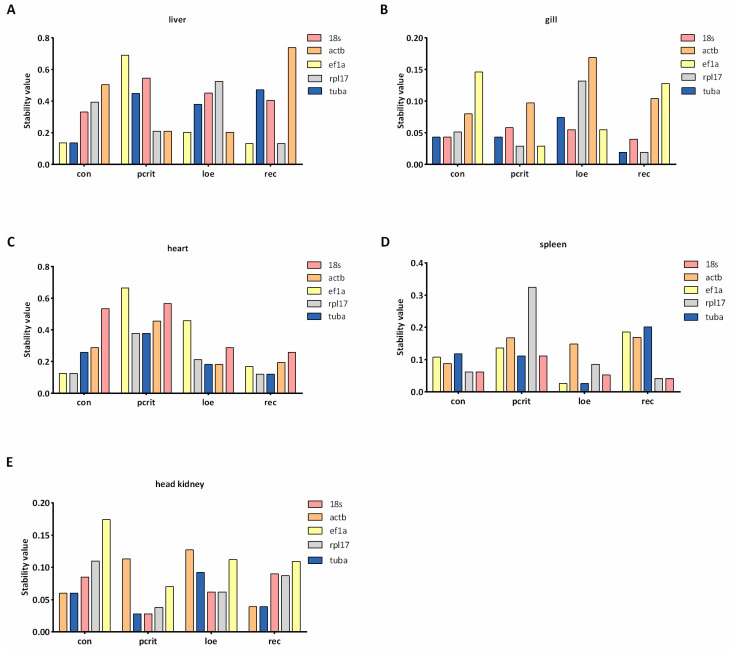
geNorm analysis of candidate reference genes’ expression stability. (**A**) The stability values of five candidate reference genes in the liver. (**B**) The stability values of five candidate reference genes in the gill. (**C**) The stability values of five candidate reference genes in the heart. (**D**) The stability values of five candidate reference genes in the spleen. (**E**) The stability values of five candidate reference genes in the head kidney. The lower stability values indicate more stable expression. con, control; pcrit, critical oxygen level; loe, loss of equilibrium; rec, recovery group.

As shown in Figure 4, the stability of candidate reference genes was analyzed by geNorm software. The results indicated that *actb* was the most stable gene in the liver under hypoxia, while *ef1a* was the common stable gene in the normal liver. In gill tissue, the most stable gene was *ef1a* in the hypoxia group, and *tuba* showed the most stable expression levels. In heart tissue, *tuba* was confirmed to be the stable gene in pcrit, loe, and rec group, and *ef1a* and *rpl17* exhibited the most stability in the control group. *Tuba* showed the most stable expression in the spleen under the hypoxia, and the *18s* and *rpl17* showed the most stable expression in normal conditions. Additionally, in the head kidney, the most common stable gene was *18s* under hypoxia, and *actb* and *tuba* were confirmed to be the most stable genes in con and rec groups.

### 3.5. Expression Stability Analysis by NormFinder

**Figure 5:**
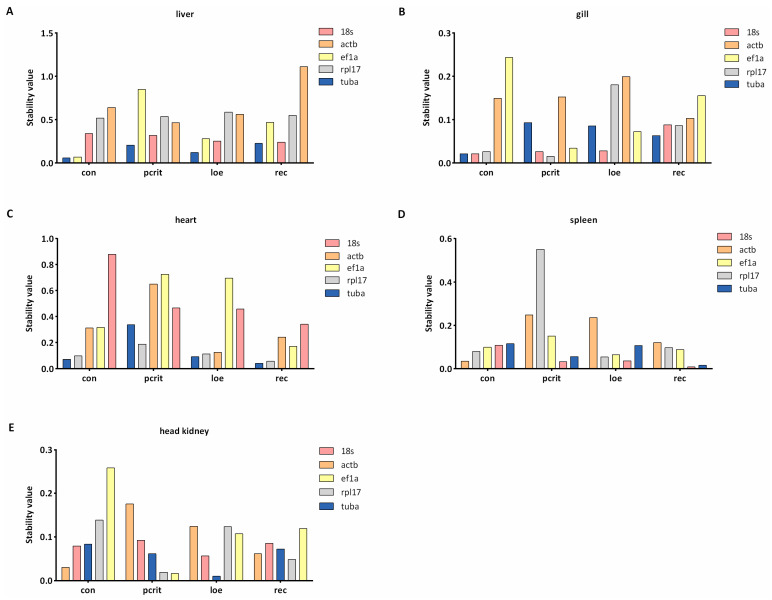
NormFinder analysis of candidate reference genes’ expression stability. (**A**) The stability values of five candidate reference genes in the liver. (**B**) The stability values of five candidate reference genes in the gill. (**C**) The stability values of five candidate reference genes in the heart. (**D**) The stability values of five candidate reference genes in the spleen. (**E**) The stability values of five candidate reference genes in the head kidney. The lower stability values indicate more stable expression. con, control; pcrit, critical oxygen level; loe, loss of equilibrium; rec, recovery group.

The stability of genes was evaluated using the NormFinder algorithm, and a low stability value indicated high stable expression. *Tuba* was observed to be the most stable gene in the liver under all conditions (Figure 5). Moreover, *tuba* and *18s* exhibited the most stability in the gills of the control group, and *18s* was identified as the common most stable gene under hypoxia conditions. *Ef1a* was the most stable gene in the heart under normal conditions, and the most common stable gene in the hypoxia group was *rpl17*. In spleen tissues, *18s* exhibited the most stability in the hypoxia and recovery groups, whereas *actb* was the most stable in the control group. In the head kidney, *ef1a* was the most stable in the pcrit group, but *tuba* exhibited the most stability in loe, and *actb* and *rpl17* exhibited the most stability in the control and recovery groups, respectively.

### 3.6. Expression Stability Analysis by BestKeeper

**Figure 6:**
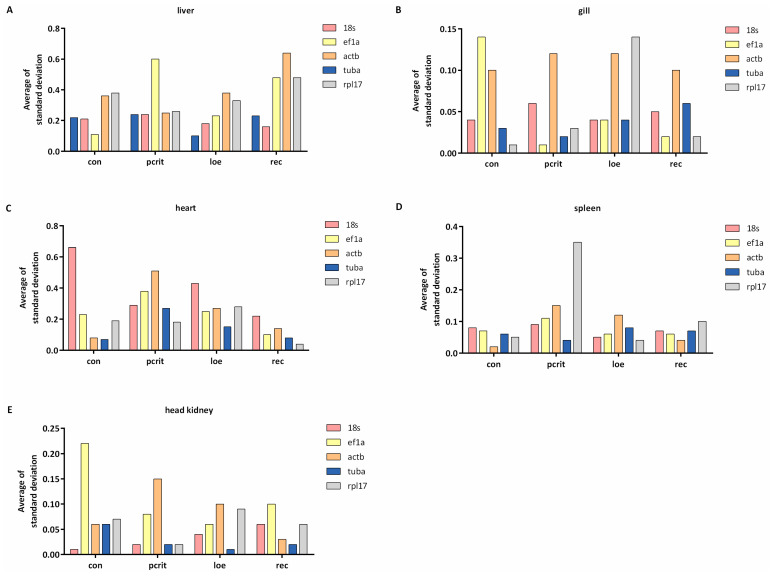
BestKeeper analysis of candidate reference genes’ expression stability. (**A**) The standard deviation values of five candidate reference genes in the liver. (**B**) The standard deviation values of five candidate reference genes in the gill. (**C**) The standard deviation values of five candidate reference genes in the heart. (**D**) The standard deviation values of five candidate reference genes in the spleen. (**E**) The standard deviation values of five candidate reference genes in the head kidney. The lower standard deviation values indicate more stable expression. con, control; pcrit, critical oxygen level; loe, loss of equilibrium; rec, recovery group.

The stability of genes was also analyzed using the BestKeeper algorithm, which ranks the reference genes by analyzing data of equivalent terminology for Ct value and Pearson correlation coefficient. Those results were visualized as Figure 6. The results show that the most stable gene in the control group was *ef1a* in liver tissues, and *tuba* was the common stable gene in the hypoxia group. In addition, *18s* exhibited the most stability in the rec group. *Rpl17* was the most stable gene in gill tissue under the normal group, whereas *ef1a* exhibited the most stability in the hypoxia group. In heart tissue, *tuba* ranked as the most stable gene in the control and loe groups, and *rpl17* exhibited the most stability in the pcrit and rec groups. *actb* was the most stable gene in the con and rec groups, and *tuba* was the most common gene in the hypoxia group. In the head kidney, *18s* exhibited the most stably expressed in the con group; however, *tuba* was expressed stably in the hypoxia group.

### 3.7. Comprehensive Analysis of Reference Genes

**Figure 7:**
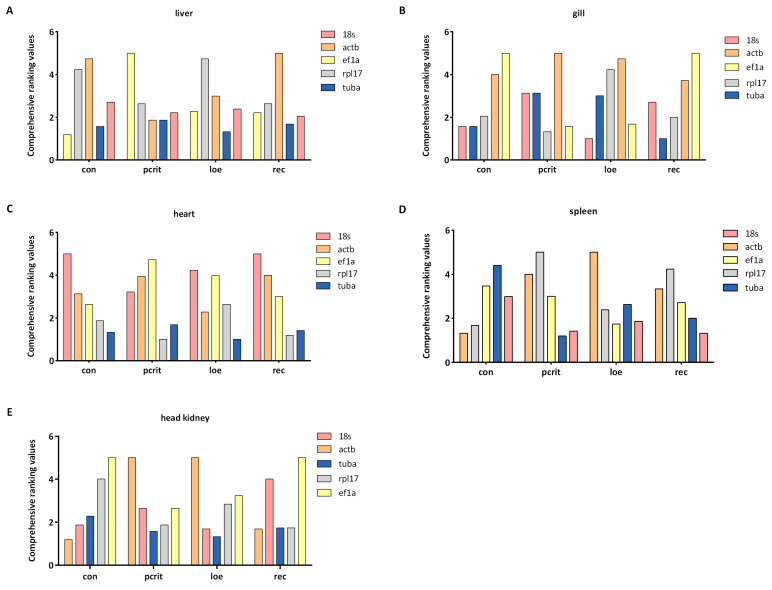
Comprehensive analysis of reference genes’ expression stability. (**A**) The comprehensive ranking values of five reference genes in the liver. (**B**) The comprehensive ranking values of five reference genes in the gill. (**C**) The comprehensive ranking values of five reference genes in the heart. (**D**) The comprehensive ranking values of five reference genes in the spleen. (**E**) The comprehensive ranking values of five reference genes in the head kidney. The lower comprehensive ranking values indicate more stable expression. con, control; Pcrit, critical oxygen level; LOE, loss of equilibrium; rec, recovery group.

In view of the differences in the calculation standards of the four methods, in order to further determine accurate results, it is necessary to carry out a comprehensive analysis by calculating the geometric mean of rankings. As shown in Figure 7, *ef1a* was the most stable gene in the liver under normal conditions, and *tuba* was the most stable gene in the hypoxia condition. Moreover, *tuba* was also stably expressed in gill tissue under normal conditions, and *18s* was the common stable gene in the hypoxia group. In heart tissue, *tuba* showed the most stability in all conditions. *Actb* exhibited the most stability in spleen and head kidney tissue under normal conditions, and *18s* exhibited stability in the spleen under the hypoxia condition. *Tuba* was confirmed to be the most common stable gene in head kidney tissue under the hypoxia condition.

## 4. Discussion

Hypoxia has significant negative effects on cultured organisms, particularly aquatic animals. When the concentration of dissolved oxygen in the water is below what is necessary for their survival, it can lead to a slowdown in growth rate, decrease in immunity, and reduction in reproductive capacity [34,35,36,37]. The changes in physiological processes are associated with the dysregulated expression of genes. RT-qPCR is one of the most important methods of revealing the mechanism of molecular regulation [38]. However, according to “MIQE” guidelines, gene expression analysis requires a stable gene as a reference, as an unstable reference gene could result in incorrect analysis. Delta Ct, geNorm, NormFinder, and BestKeeper software rely on Ct values or expression-level data derived from RT-qPCR. Each employs distinct algorithms to compute the stability metrics of reference genes, and these tools are currently the most widely utilized approaches for assessing gene expression stability [39,40,41].

In general, housekeeping genes and ribosomal protein genes are often selected as internal reference genes due to their stable expression. *Actb*, *ef1a*, *tuba*, *rpl17*, and *18s* were often used as internal reference genes in previous studies on fish, so we selected these genes to analyze whether they are stably expressed under hypoxic conditions [14,42,43]. *Actb* is a key protein constituent of striated muscle fibers, and it also plays a significant role in muscle filaments and the microfilament structure of the cytoskeleton [44]. *Ef1a* is one of the most abundant factors in protein synthesis, with highly conserved genetics and expression [45]. *Rpl17* is a conserved gene related to ribosome synthesis [46]. The *18s* rRNA gene is a DNA sequence encoding small subunits of eukaryotic ribosomes [47], and the *tuba* gene encodes the microtubule protein, which is a skeletal protein of cells and is widely distributed [48].

In the previous studies, Jiang et al. found that *actb* was expressed stably in the head kidney of Oreochromis Niloticus [49]. Moreover, *ef1a* and *actb* were validated to select the best internal control for qPCR in early ontogenesis of *Salmo salar* [50]. *Rpl17* and *ef1a* exhibited high stability in the liver of black rockfish [22]. Tang et al. reported that *18s* exhibited stable expression in the gene expression studies for zebrafish [51]. *Ef1a* was used as a reference gene in the hypoxia study of black rockfish by analyzing and comparing Ct values [52]. However, Zheng et al. reported that *18s* was recognized as the reference gene for gene expression in black rockfish [43]. Although the expression of these genes has been analyzed, those results have only been applicable to certain tissues and their expression under hypoxic conditions has not been detected, highlighting the need for careful tissue-specific selection of reference genes under hypoxic stress.

In this study, our analysis under hypoxic conditions revealed significant insights into the regulation of housekeeping genes, which are typically assumed to be unchanging across diverse cellular environments. Comprehensive analysis results showed that *tuba* had the best stability across all tissues and conditions. *Tuba* was stably expressed in normal liver, heart, and gill tissues, and it was also the most stable under hypoxic conditions in the liver and head kidney. Under hypoxic conditions in gill tissue, *ef1a* was the commonly expressed most stable gene. In hypoxia studies of the heart, *rpl17* and *tuba* could be used as reference genes. In spleen tissue, *18s* showed very stable expression and could serve as a reference gene under hypoxic conditions. However, under normal conditions, *actb* was the most stably expressed in spleen and head kidney tissue. Our results have some similarity to previous studies mentioned above, but here, we used multiple methods to comprehensively analyze and compare the expression stability in five tissues of the black rockfish. Due to the selection of these five candidate genes in our previous research, there was not a significant difference between the results. Overall, *tuba* offers a more significant advantage when choosing reference genes for the black rockfish.

In conclusion, our findings highlight the critical need to reassess the choice of internal control genes in studies related to hypoxia to guarantee the precision of experimental results. This re-evaluation is pertinent not only to cellular hypoxia research but also extends to any field where gene expression analysis is performed under non-normoxic conditions. Nonetheless, further studies are warranted to corroborate the findings of this research.

## Figures and Tables

**Table 1:** The information on primers in this study.

Gene Name	Sequence	Tm (°C)	Accession Number	Product Length
*18s*	F: CGGTCGGCGTCCAACTTCTTAG	65.8	KF430619	144
	R: TCTCGGCGAAGGGTAGACACAC	63.9		
*actb*	F: AGAGGGGTTACAGCTTCACC	55.8	KF430616	140
	R: CTCGTAGCTCTTCTCCAGGG	56.6		
*ef1a*	F: GCGGAGGCATCGACAAGAGAAC	65.8	KF430623	89
	R: CAGCACCCAGGCGTACTTGAAC	64.4		
*tuba*	F: GGTGGCTGGTAGTTGATG	50.7	KF430618	110
	R: GTGCCCAAAGATGTGAAT	50.2		
*rpl17*	F: AGGCGACGCACCTACCG	55.5	KF430620	109
	R: CCTCTGGTTTGGGGACGA	55.9		

F: forward primer, R: reverse primer.

## Data Availability

The original contributions presented in the study are included in the article, further inquiries can be directed to the corresponding author.
